# Developing an implementation strategy for a digital health intervention: an example in routine healthcare

**DOI:** 10.1186/s12913-018-3615-7

**Published:** 2018-10-19

**Authors:** Jamie Ross, Fiona Stevenson, Charlotte Dack, Kingshuk Pal, Carl May, Susan Michie, Maria Barnard, Elizabeth Murray

**Affiliations:** 1 Research Department of Primary Care and Population Health, UCL, Upper 3rd floor, Royal Free hospital, Rowland Hill St, London, NW32PF UK; 2 Department of Psychology, University of Bath, Bath, UK; 3 Faculty of Health Sciences, University of Southampton, Southampton, UK; 4 Health Psychology, UCL, London, UK; 5 Department of Diabetes & Endocrinology, Whittington Health NHS Trust, London, UK

**Keywords:** Implementation, Implementation planning, Implementation strategy, Implementation theories, Digital health, Routine practice, Healthcare, Diabetes mellitus, Type 2, Health plan implementation, Delivery of health care

## Abstract

**Background:**

Evidence on how to implement new interventions into complex healthcare environments is often poorly reported and indexed, reducing its potential to inform initiatives to improve healthcare services. Using the implementation of a digital intervention within routine National Health Service (NHS) practice, we provide an example of how to develop a theoretically based implementation plan and how to report it transparently. In doing so we also highlight some of the challenges to implementation in routine healthcare.

**Methods:**

The implemented intervention was HeLP-Diabetes, a digital self-management programme for people with Type 2 Diabetes, which was effective in improving diabetes control. The target setting for the implementation was an inner city London Clinical Commissioning Group in the NHS comprised of 34 general practices. HeLP-Diabetes was designed to be offered to patients as part of routine diabetes care across England. Evidence synthesis, engagement of local stakeholders, a theory of implementation (Normalization Process Theory), feedback, qualitative interviews and usage data were used to develop an implementation plan.

**Results:**

A new implementation plan was developed to implement HeLP-Diabetes within routine practice. Individual component strategies were selected and developed informed by Normalization Process Theory. These strategies included: engagement of local opinion leaders, provision of educational materials, educational visits, educational meetings, audit and feedback and reminders. Additional strategies were introduced iteratively to address barriers that arose during the implementation. Barriers largely related to difficulties in allocating resources to implement the intervention within routine care.

**Conclusion:**

This paper provides a worked example of implementing a digital health intervention. The learning from this work can inform others undertaking the work of planning and executing implementation activities in routine healthcare. Of particular importance is: the selection of appropriate theory to guide the implementation process and selection of strategies; ensuring that enough attention is paid to planning implementation; and a flexible approach that allows response to emerging barriers.

**Electronic supplementary material:**

The online version of this article (10.1186/s12913-018-3615-7) contains supplementary material, which is available to authorized users.

## Background

Many examples of problematic implementation of health interventions within healthcare settings exist [1–5]. It is increasingly recognised that the manner in which interventions are implemented is as important to realise anticipated benefits as are the features and functions of the interventions themselves [6]. Implementation strategies have been referred to as the ‘how to’ of implementation science [7] comprising the specific means or methods for adopting and sustaining interventions [8]. A well designed implementation plan may be particularly important for the implementation of complex interventions [9], meaning interventions “consisting of multiple behavioral, technological, and organizational components” [10]. The interplay between these various parts is often non-linear and unclear, making the implementation and evaluation inherently difficult [9].

Digital health interventions are one example of complex interventions that have proved difficult to implement due to factors such as interoperability, cost, fit with existing systems, disruption to interactions between health professionals and patients, and poor implementation planning [5, 11, 12]. Given the increasing use of digital interventions for healthcare provision, understanding how best to implement them is crucial. Despite the importance of planning implementation, descriptions in the literature of specific implementation strategies used are sparse, poorly reported and indexed [13]. Insufficient detail on the context and implementation process can hamper replication and scale-up of interventions, may contribute to the gap between research and practice [14, 15] and makes it difficult for implementation researchers and other stakeholders to fully utilize the findings of studies [7]. Specific criticisms of the reporting of implementation studies include poor (or absent) descriptions of conceptual frameworks underpinning the research, inadequate description of context, and incomplete information about the implementation plan itself [13, 16, 17]. It can be argued that in an implementation study, it is the implementation plan that is the intervention of interest, and hence the arguments for publishing descriptions of such plans are similar to those for publishing descriptions of complex or behavioural interventions [18]; that in order to advance the science of implementation, clear and transparent reporting of implementation methods is needed [19, 20].

In this paper we describe the methods of developing an implementation plan (used in this context to describe a multifaceted approach comprised of component implementation strategies e.g. audit and feedback) to implement a digital health intervention within routine clinical practice. It is hoped that by transparently describing the process by which the plan was developed, the strategies selected to affect change and the adaptations to the plan, this will provide an example for others undertaking implementation work of how to develop and report implementation activities as recommended [13, 21]. By describing the adaptations that were made to the implementation plan once it was deployed in practice, we highlight some of the challenges to implementing digital health interventions into routine NHS practice. This is an increasingly important area for study, given the policy focus on use of digital health interventions [22].

### The problem addressed by the digital health intervention

The prevalence of Type 2 Diabetes (T2DM) is increasing globally [23] and a key strategy for managing the health care of people with diabetes is self-management [24]. In the UK, education to support people with T2DM to manage their condition is recommended by the National Institute for Health and Care Excellence (NICE) [25]. Figures from the National Audit Office suggest that this education, usually delivered face to face, is poorly attended by patients, with only 8.2% of eligible patients attending [26]. Issues with accessibility, anonymity and convenience have been suggested as reasons for this low uptake [27–29]. In response to this, and the growing need to find alternative ways of delivering healthcare [2], a digital solution for delivering diabetes education was developed.

### The intervention

HeLP-Diabetes (Healthy Living for People with Type 2 Diabetes) is a digital self-management intervention for people with T2DM which has been shown to improve glycaemic control over 12 months [30] and be cost-effective compared to usual care [31]. The design of this complex intervention is reported by Dack et al. [32]. In summary, HeLP-Diabetes is an online programme that can be accessed on desktop computers, tablets, and mobile devices with an internet connection. It addresses a wide range of patient needs including education, lifestyle changes, medicine management, emotional management, and social support. The information provided on HeLP-Diabetes is based on NICE guidelines and has been developed to complement existing face-to-face structured group education which includes programmes such as DESMOND [33] and Co-creating health [34]. The development of HeLP-Diabetes was informed by theory and the needs and preferences of patients and health professionals, developed using a process of participatory design. HeLP-Diabetes was designed to be offered to patients during routine NHS appointments and a degree of facilitation from staff to help patients register to use the intervention was considered part of the intervention.

This research was conducted in parallel with a randomised control trial of the HeLP-Diabetes intervention to assess effectiveness and cost-effectiveness [30] and aimed to provide complementary data on how best to implement the intervention in practice by developing an implementation plan and collecting data on: the adoption of the intervention by health services; the uptake and use of the intervention in an unselected patient population in routine care; factors that inhibit or facilitate implementation into existing NHS services; determine the resources needed for effective implementation. This paper focuses specifically on describing the methods used to develop the implementation plan which was designed to integrate HeLP-Diabetes within routine practice. Other papers focus on the design of the implementation study more broadly [35], the development [32], effectiveness [30] and cost effectiveness of the HeLP-Diabetes intervention [31].

### Theoretical approach

Normalization Process Theory (NPT) [36, 37] was selected to guide the development of the implementation plan. A greater use of explicit theory in order to understand barriers and facilitators, select implementation strategies and design interventions, has been advocated to advance implementation research [7, 20]. The use of theory in implementation also allows for easier replication of successfully implemented interventions [38]. In order to select the most appropriate theory, we developed a set of criteria based on a taxonomy by Tabak et al. [38]. The criteria were that the selected theory should: 1. focus on implementation (rather than dissemination- a more passive process) 2. be applicable to individual and organization levels of implementation (as the implementation of HeLP-Diabetes was likely to involve change at both these levels) 3. be a broad theory of implementation (rather than a process theory) which could help plan implementation, identify barriers and facilitators to implementation, guide the selection of strategies to effect change and help evaluate and explain the success of the implementation. 4. have been used successfully in relation to digital health interventions and in the healthcare setting. Sixty theories identified in a systematic review of implementation theories [38] were assessed against these criteria (Additional file 1 presents this assessment) (Co-author May was not involved in this selection process due to the conflict of interest this would have posed). NPT satisfied all these criteria and was therefore selected to underpin our theoretical approach.

NPT is a widely used [39, 40] theory of implementation that can be used to explain the processes by which an intervention becomes, or indeed fails to become, normalised into routine practice. It offers a framework for assessing the conditions in which interventions become practically workable in healthcare. Normalization is defined as the embedding of a technology as a routine and taken-for-granted element of clinical practice [41] and focuses on the ‘work’ of implementation. This is represented by four constructs: *Coherence*: the work that people do to understand and make sense of a practice; *Cognitive participation*: the work that people do to engage and support a new practice; *Collective action*: the work that people do to enact a new practice, and make it workable and integrate it in its context; and *Reflexive monitoring*: the work that people do to reflect on and evaluate enacting a new practice in context. The collective action construct has four important areas: *interactional workability* which is the impact of the intervention on consultations, *relational integration* which is the impact on relationships between professionals, *skill set workability* which is the fit with existing skill sets and responsibilities and *contextual integration* which is the fit with organisational priorities and resources. Normalization of an intervention into routine practice is more likely if there is positive coherence, cognitive participation, collective action and reflexive monitoring. Throughout the development of the implementation plan we considered all of the main constructs of NPT: coherence, cognitive participation, collective action and reflexive monitoring, and where appropriate, also the subconstructs of each of these (see Additional file 2 for a description of constructs and subconstructs provided by the NPT online manual and toolkit [42]).

### Approach to implementation

We also drew on principles derived from the work of Grol and Wensing who provide a systematic approach to implementation planning and execution [43]. This work was drawn upon as a guide to how to approach designing an implementation plan. This specific model was selected because it was a process model that provides clear, easy to follow steps, the authors are leaders in the field of implementation science, it applies directly to thinking about the implementation of complex interventions within healthcare and has been applied widely in other implementation studies (see for example [44–47]). They highlight that in order accomplish implementation within healthcare practice: (i) attention must be given to the development of concrete proposals/targets for improvement or change, (ii) it is important to conduct an analysis of performance, target group and setting, (iii) the selection of implementation strategies should be appropriate and evidence based, (iv) there should be development, testing and execution of an implementation plan, (v) evaluation and adaptions to the plan may be necessary. We applied these principles to our thinking about how to plan, organise and execute the implementation of HeLP-Diabetes by operationalising them into a set of specific objectives for the development of the HeLP-Diabetes implementation plan (described below) and to inform our thinking about how to execute the implementation plan.

### Aims and objectives

This paper aims to clearly report the development of an implementation plan to implement a complex digital health intervention (HeLP-Diabetes) within primary healthcare services.

The specific objectives are to:

1. understand barriers and facilitators to implementing digital health interventions
2. develop a thorough understanding of the implementation context
3. use this understanding to select theoretically informed and evidence based implementation strategies and develop them into an implementable plan
4. execute, evaluate and make adaptations to the implementation plan

## Methods

**Fig. 1:**
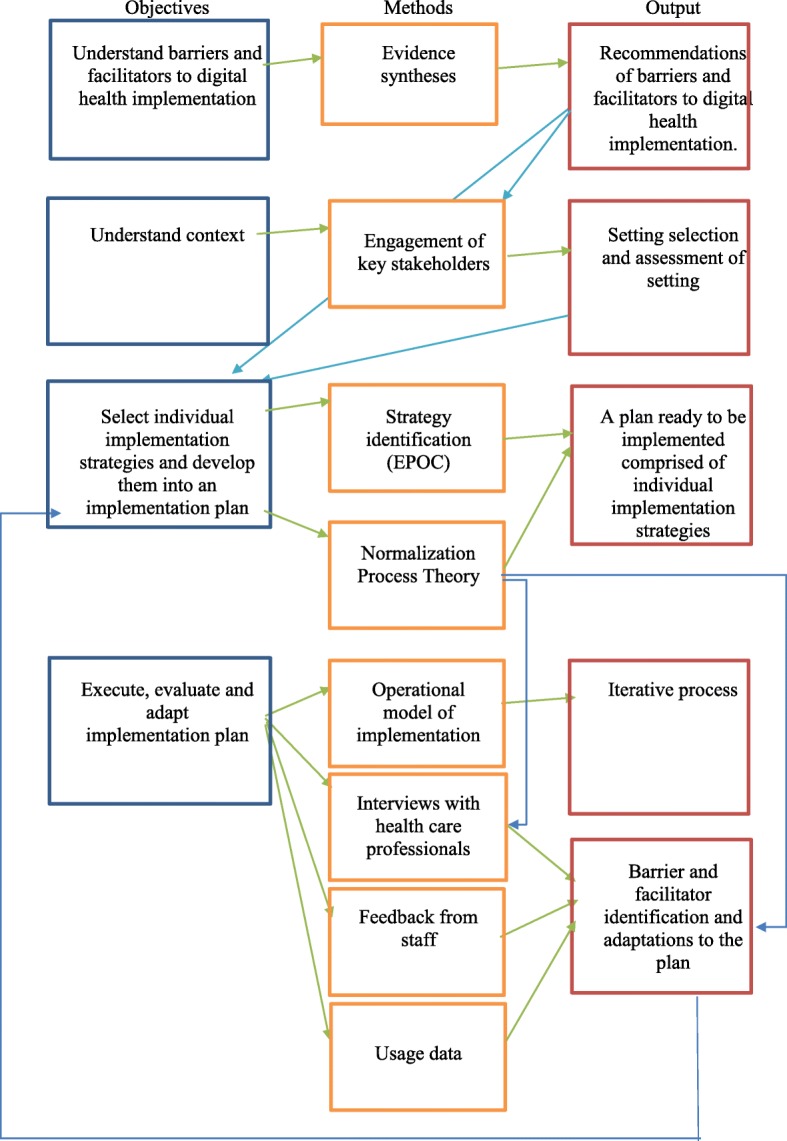
Methods used to develop an implementation plan for the HeLP-Diabetes intervention

See Fig. 1 for an overview of these methods.

Objective 1: understand barriers and facilitators to implementing digital health interventions.

### Evidence synthesis

We undertook a systematic review of the literate on factors that influence the implementation of e-health (see Ross et al. for detailed methods [5]) to identify barriers and facilitators pertinent to implementing e-health. In summary, this review identified forty four papers using search terms related to implementation and e-health (which we now describe as digital health), data were extracted and synthesised according to a framework of implementation (the Consolidated Framework for Implementation Research [48]) which categorises implementation factors as relating to the intervention; the inner context; the outer context; the individual; or the process of implementation. The main findings from this review that we drew on to inform the development of the implementation plan were:

- The need for careful consideration of the effects of the intervention on existing systems and work practices
- Key stakeholders and implementation champions should be included as early as possible in the implementation process
- Planning implementation is a critical step which includes ensuring that organisations are in a state of readiness.
- The provision of training and education to all those involved with implementation is a key success factor
- Implementation does not stop with ‘go-live’—there is a need for ongoing monitoring, evaluation and adaptation of systems to ensure intended goals are being met, benefits realised, and ongoing identification of barriers to effective use, along with strategies to overcome these barriers.

These findings were used to inform our selection of appropriate individual strategies to implement HeLP-Diabetes (such as training and the involvement of key stakeholders) but also to guide the way in which we conducted the implementation process (i.e. taking into account the need for ongoing monitoring, evaluation and adaptation). There were other findings from this review that we did not action as they were either not relevant in this context or because we were unable to influence them (for example; one finding highlighted the need for legislative support for e-health technologies which we were unable to address given the limits of our research scope).

Objective 2: develop a thorough understanding of the implementation context.

### Engagement of key stakeholders

The systematic review highlighted the importance of engaging key stakeholders early in the implementation process. A steering group comprised of health professionals (GPs, Consultants, Diabetes Specialist Nurses, Dieticians and Psychologists) working in diabetes care was established before the beginning of the implementation to support the research team and provide insight into how diabetes care is organised within the NHS and how diabetes education is currently offered and provided to patients. Early engagement with a Clinical Commissioning Group Officer (from the CCG later chosen to implement the intervention in) provided further contextual information about diabetes care in the primary care setting (which had been selected as the broad setting prior to intervention development). This analysis culminated in the following learning points about how T2DM care was organised and provided by primary care services within the NHS.

- Referral to diabetes education was recommended by NICE for all patients newly diagnosed with type 2 diabetes
- Referrals to this education for patients with T2DM were provided mainly through primary care setting
- The national figures (at the time of the study) of the numbers of referrals made to education were very low (only 11.5% of people with T2DM were being offered structured education (with 5.6% actually attending) [49]
- Referring to education was part of current practice for health professionals working in primary care and therefore referring to HeLP-Diabetes was a recognised and accepted practice.
- However, using HeLP-Diabetes would require an entirely new set of practices to be enacted by healthcare professionals (the concrete targets for change for primary care practices and staff are detailed in Table 1).

**Table 1:** The concrete targets for change for primary care practices and staff with the implementation of HeLP-Diabetes

Practice targets: • To adopt HeLP-Diabetes as an additional service for their patients with T2DM, • Provide resources (time, healthcare professionals, and space) to allow staff to offer the intervention to patients	
Healthcare professional targets: Behaviours additional to normal practice were required to: • Recommend HeLP-Diabetes to patients during routine appointments • Register patients (or assist patients to register) to use HeLP-Diabetes, either within routine appointments or at a separate time • Facilitate patient use of HeLP-Diabetes through a facilitation appointment where key features of HeLP-Diabetes are shown to patients by staff • Encourage ongoing use of HeLP-Diabetes in patients by discussing use in subsequent appointments.	

#### Setting selection

This analysis confirmed that primary care was the most appropriate setting for HeLP-Diabetes to be implemented given that the majority of self-management discussions, T2DM care provision and referrals to other services are provided through primary care. The scope of the research study limited the research to one Clinical Commissioning Group (CCG). CCGs are membership bodies, with local GP practices as the members, and they are responsible for the planning and commissioning of health care services for their local area. It is common for CCGs to commission a new service and encourage individual practices to use it, therefore the implementation was targeted at the CCG as a whole and the individual practices within. It was believed, based on findings from the evidence synthesis that having CCG support for HeLP-Diabetes would be important for enhancing credibility and, in NPT terms, likely to promote coherence and cognitive participation. To a large extent, the outer context (national priorities, policies and economic incentives) of the implementation was outside the researchers’ control, so it was important to select a CCG where the inner context was likely to be favourable, and where it would be reasonably easy to study the implementation (as this was a pragmatic exploratory study concerned with how best to implement HeLP-Diabetes). The selection of the CCG was based on the following criteria:

- Diabetes should be a local priority for the CCG;
- There should be interest in promoting self-management by patients;
- It should be reasonably local to the research team, since implementation is promoted by good communication and local ownership (as identified by the systematic review);
- The CCG should be interested in working with the research team.

Once the CCG was selected, in depth (informal) discussions with key stakeholders within the CCG informed our understanding of the context further. The main learning points from the assessment of the specific setting were:

- There were 34 GP practices within the CCG
- Within the CCG self-management of T2DM was high on the agenda
- There was a specific diabetes working group within the CCG comprised of GPs
- Many practices had a lead for diabetes (usually a GP)
- Much of the work within practices around self-management was the responsibility of nurses
- Most practices had clinical meetings at lunchtime where they were used to new working practices being introduced to them

Objective 3: select theoretically informed and evidence based implementation strategies and develop them into an implementable plan.

### Selection of implementation strategies

We identified potential strategies from the Cochrane Effective Practice and Organisation of Care (EPOC) taxonomy of implementation strategies [50] and used evidence gathered from the previous stages; the evidence synthesis and the assessment of the implementation context, to inform the selection of the most suitable strategies to implement HeLP-Diabetes. We also based our selection on NPT, choosing strategies that we thought would bring about change based on increasing coherence, cognitive participation, collective action and reflexive monitoring. The individual strategies selected, and the constructs of NPT that they target are presented in Table 2 in the results section.

Objective 4: Execute, evaluate and make adaptations to the implementation plan.

#### Execution

The systematic review, and principles from Grol & Wensing’s model of implementation guided the decision to make the implementation process iterative in two ways. Firstly, Grol and Wensing recommend starting implementation on a small-scale and testing strategies on a modest sized motivated group before moving on to larger populations. As such, the intention was for the first iteration of the implementation plan to be tried out on a small batch of GP practices within the CCG. Following the implementation of HeLP-Diabetes in this first batch of practices, a staged roll out was then planned for the remaining services, whereby the implementation plan would be targeted at another few practices at a time, and then another few, as opposed to a widespread implementation targeted at all practices at once. The aim of the staged roll out was to learn from the experience of implementing on a small scale and apply this learning to adapting strategies for implementation at subsequent practices and to avoid implementing unsuccessful strategies across all practices.

Secondly, the systematic review and the process model stress the importance of continually evaluating the implementation and making adaptations to the plan if needed. Data were collected in several ways to inform our evaluation of the implementation. Throughout the implementation phase, barriers to implementation were identified by staff through informal feedback discussions as well as through formal data collection using qualitative interviews and intervention usage data. Written consent was obtained from all participants taking part in interviews and to access anonymous usage data.

### Feedback

The researcher who was leading the implementation within the CCG (co-author Ross) was in regular contact with staff at GP practices that were involved in implementing HeLP-Diabetes. These encounters with staff provided opportunities for them to feedback informally to the researcher about the implementation process. During these discussions, staff discussed barriers that were arising and also provided suggestions on how the implementation could be improved. A log of the salient points of these discussions was kept by the researcher and fed back into the iterative implementation plan.

### Interviews

Interviews were conducted with 21 health professionals throughout the course of the implementation (detailed methods are to be reported separately). The sample were selected purposively and comprised staff who worked within practices where HeLP-Diabetes had been offered for use. Participants included GPs, practice nurses, diabetes specialist nurses, health care assistants, administrative and reception staff, practice managers and commissioners of care. Interview topic guides were informed by NPT and explored the implementation of HeLP-Diabetes in NHS practice. Interviews were audio recorded and transcribed verbatim. The data were first analysed thematically and then themes were mapped onto constructs of NPT. Although the data analysis was not completed until after the implementation study had finished, data provided during the interviews that related to barriers and facilitators to the implementation were captured and fed back into the iterative implementation plan.

### Usage data

HeLP-Diabetes software (Joomla) recorded the number of patients signing up to HeLP-Diabetes and the GP practices that they were registered at. The researcher (co-author Ross) checked these figures weekly and used the data to determine how each practice was performing. For example, if patients were regularly being registered to use the intervention from a particular practice it was assumed that staff were implementing it well. However, if there were no patients being signed up, this gave an indication that there may be a problem with the implementation. This monitoring prompted contact to be made with staff in these practices where there seemed to be problems and for feedback from staff to be collected.

#### Adaptations

Feedback from staff, interview data and usage data provided an ongoing evaluation of the implementation and identified barriers (described in the results section). As an iterative study, in cases where it was possible, these barriers were tackled by making modifications to the implementation plan. Ineffective strategies were removed and new strategies incorporated.

## Results

**Fig. 2:**
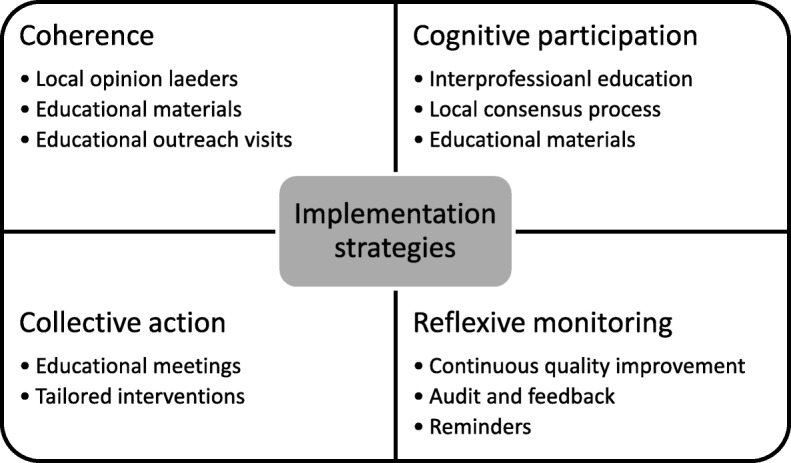
Implementation strategies selected to target constructs of NPT

An executable implementation plan was developed informed by a systematic review of the literature, a theory of implementation and data derived from staff feedback, qualitative interviews and usage data collected from the intervention. Individual implementation strategies were selected to target the constructs of NPT and thus make implementation more likely (presented in Fig. 2). These strategies were combined and operationalised for the HeLP-Diabetes intervention within the implementation plan which is presented in Table 2 and summarised below.

**Table 2:** The HeLP-Diabetes implementation plan

Strategy	Strategies operationalised for HeLP-Diabetes
To target coherence:	
Local opinion leaders	• Key people within the CCG were identified at a CCG local policy meeting. • All practice managers and lead GPs for diabetes were informed about HeLP-Diabetes by email
Educational materials	• Information email sent to all practice managers and leads emphasised that HeLP-Diabetes was an online programme thus different from other self-management programmes, that it was free to use and had been developed by a university. • Flyers, posters and other advertising materials were developed and circulated throughout the CCG.
Educational outreach visits, or academic detailing	Meetings were arranged between practices and the research team to provide health care professionals (HCPs) with information about HeLP-Diabetes and discuss the implications for their working practice, in order to allow them to decide whether or not to adopt it. Informed by the need to promote coherence (sense-making), during these meetings we emphasised the online nature of the programme, its evidence-base, theoretical underpinning and participatory design, and the potential benefits to patients, practices and the healthcare system.
To target cognitive participation:	
Educational meetings	We promoted cognitive participation during meetings at practices by emphasising the benefits to patients, practices and healthcare system (coherence), while ensuring minimum workload and optimal fit with interactional workability, skill set workability, contextual integration and relational integration (Collective Action).
Inter-professional education	HCPs were provided with a training session which provided the opportunity for staff to understand the actions and procedures needed to sustain HeLP-Diabetes in practice and see that HeLP-Diabetes could deliver the anticipated advantages.
Local consensus processes	Training was with groups of staff which allowed the opportunity for them to discuss and decide how the work of implementing would be shared within the practice and how HeLP-Diabetes would be offered to patients.
To target collective action:	
Educational meetings	Staff were provided with login details which allowed them to try out HeLP-Diabetes. This allowed staff to see how HeLP-Diabetes fitted with the skill sets of the HCPs in the practice (skill set workability), what resources were needed to make it part of routine practice (contextual integration), what knowledge was needed to be confident with HeLP-Diabetes as a new way of working (relational integration), and the impact that HeLP-Diabetes would have on interaction with colleagues and patients (relational integration).
Educational materials	Training booklets were developed and provided to staff at the training sessions containing information on how to access HeLP-Diabetes, how to create a login, and how to sign patients up and provided summaries of the different parts of the intervention and how to use them with patients.
To target reflexive monitoring:	
Continuous quality improvement	• Ongoing support and communication was provided to each service who adopted HeLP-Diabetes to allow problem solving and maintain awareness of HeLP-Diabetes. • Data on the number of patients being registered at each practice was collected • Informal discussions and interviews with staff were conducted in order to identify barriers to the implementation and to develop solutions.
Audit and feedback	Feedback that included number of patients using HeLP-Diabetes, how each service was performing and feedback from patients using HeLP-Diabetes was provided to services via email regularly to promote positive reflexive monitoring.
Reminders	Regular emails and newsletters were sent from the research team and the CCG to practices to remind them about HeLP-Diabetes and to encourage those who had already adopted it to keep referring patients to use it.

We targeted the concept of coherence (sense-making) through emphasising the on-line (as opposed to face-to-face) nature of HeLP-Diabetes, the theoretical underpinning, evidence-base and participatory design principles that had contributed to its development, and the likely benefits to patients in a series of key meetings with local opinion leaders and practice outreach visits as well as in our publicity material. Cognitive participation (deciding whether to participate in the work of implementation) was targeted through emphasising the benefits to patients, practices and the health care system, while minimising the work required of healthcare professionals. Collective action (the work of implementation) was addressed through developing and delivering training materials and training sessions, and adapting the implementation processes to reduce healthcare professional workload. Reflexive monitoring was addressed through ongoing support and communication with the practices, including feedback on the numbers of patients registered, reminders, and rapid responses to any barriers identified.

The barriers that were identified to implementation were considered using NPT constructs in order to provide explanations and to identify strategies to overcome them. Strategies to target these barriers were aimed at increasing coherence, cognitive participation and collective action and providing opportunities for positive reflexive monitoring. These barriers and the strategies selected to overcome them are presented in Table 3.

**Table 3:** Barriers identified to the implementation and strategies employed to address them

Barrier	Strategy to address barrier	Strategies operationalised for HeLP-Diabetes
Collective action (contextual integration)		
Staff unwilling or unable to provide the resources to implement the facilitation aspect of the registration process (see Table 1).	Tailored intervention	For practice who identified a lack of resources to implement HeLP-Diabetes a streamlined process which removed the facilitation aspect was offered. of the process.
Even after removal of facilitation aspect some practices still couldn’t find resources to register patients	Tailored intervention	Alternative patient registration methods including patient self-registration and peer supported were offered to practices.
Collective action (Skill Set Workability)		
Nurses, who had originally been targeted to deliver the intervention, felt that the using a digital intervention underutilized their own knowledge about diabetes. Health Care Assistants with additional knowledge.	Tailored intervention	Health Care Assistants were targeted to deliver the as they were often younger, IT literate, keen to help patients, but knew there were limitations to their diabetes knowledge that the intervention could help provide them.
Cognitive participation		
Some staff reported not remembering or having other competing priorities which prevented HeLP-Diabetes being offered to patients.	Reminders	To keep the new way of working in view and connect it to the people who needed to be doing the work, HeLP-Diabetes was integrated within practice templates which prompted staff during appointments with patients with T2DM to mention HeLP-Diabetes and provide a leaflet.
Collective action (relational integration)		
Some staff were unaware of HeLP-Diabetes within practices where adoption had been agreed. This was often due to teams not communicating about HeLP-Diabetes or in several cases because those who made adoption decisions (usually GPs) were not the ones tasked with implementing it.	Educational meetings and materials	To increase the visibility of HeLP-Diabetes additional staff focussed advertising was introduced including exhibition stalls, talks and demonstrations at staff education events. HeLP-Diabetes was also frequently advertised in the CCG’s bulletin to GPs. HeLP-Diabetes was also included by the CCG as one of their Locally Enhanced Services and added to the Map of Medicine system used by GP practices in the CCG.
Reflexive monitoring impacting on interactional workability		
Staff suggested that they would offer HeLP-Diabetes to patients more if they were receiving more requests or enquiries from patients about it.	Patient-mediated interventions	Additional patient focussed advertising strategies were introduced to promote HeLP-Diabetes to increase the requests/enquiries from patients about HeLP-Diabetes. These included TV screen adverts in waiting rooms, talks given at patient self-management groups, attendance at Diabetes UK events, coverage in Practice newsletters and a mass mail out to all patients in some practices.

### Analysis of the implementation plan

Interpreting the barriers and facilitators to implementation through a NPT lens helped us to explain the implementation of a digital health intervention within routine NHS practice. This analysis showed that while there seemed good understanding about HeLP-Diabetes and its value (coherence), difficulties arose with staff mobilising time and effort to do the work required to make HeLP-Diabetes integrated into practice (collective action). The registration process was one of the most widely reported barriers and there was a lack of agreement by staff (collective action: relational integration) as to whether this was a legitimate part of their role (collective action: skill set workability) with some reporting that it wasn’t a suitable use of time or resources (cognitive participation). Staff reported that their participation might increase had they more opportunity to reflect on the worth of the intervention (reflexive monitoring) through feedback from patients.

### Adaptations to the implementation process

During the study some adaptations were made to the process of implementing the plan.

### Changes to the target setting

Initially, the implementation plan had focused on GP practices only. However, during an early steering group meeting, it was decided that to fully integrate HeLP-Diabetes into routine NHS care it should be made available to patients through a range of settings. The target settings were therefore extended to include hospital and community diabetes clinics (as well as GP practices).

### Changes to the execution of the implementation plan

In securing support from the CCG to promote the roll out of HeLP-Diabetes across the CCG to all practices, this changed the planned batch roll out (see above). The CCG wanted a mass roll out with HeLP-Diabetes being made available to all practices as quickly as possible. This changed the iterative approach to the execution of the plan and took some of the control of the implementation away from the research team and placed it with the CCG.

## Discussion

An executable implementation plan was developed informed by a systematic review of the literature, a theory of implementation and data derived from staff feedback, qualitative interviews and data about use collected from the intervention to implement a digital health intervention within routine NHS services. Often reporting of implementation planning is sparse and there are few examples in the literature of how to develop implementation strategies. This paper transparently described the process of developing and implementing this plan and provides a clear example to others undertaking implementation in routine healthcare. We have also generated learning on some of the barriers and facilitators to implementing digital health interventions within routine NHS practice. We found that requiring staff to provide support to patients to register to use a digital health intervention was a barrier in such a resource tight NHS. We also showed that nurses did not perceive using HeLP-Diabetes a legitimate part of their role and that other members of practice staff such as HCA’s might be better suited to this role. Finally, providing feedback to staff from patients about the value they get from the intervention is likely to increase motivation to continue engaging with it.

### Strengths and weaknesses of the study

Conducting this research within the parameters of a research study led to several challenges. In line with gaining ethical approval to conduct research within the NHS, the target setting for the implementation had to be identified before the start of the study and before a formal appraisal of the primary care context could be completed. Hindsight suggests that the implementation of a new intervention at the time the study was conducted was always likely to encounter many barriers. During the study period, the NHS was described as being in ‘crisis’ [51] with increased complexity in patient cases, reduced workforces and primary care budgets, GP time being spent on duties other than care giving, and political and public pressure on GPs for increased access to services [52–54]. This period saw the closure of many GP practices [55, 56] and in fact, three GP practices within the study CCG closed down during the implementation of HeLP-Diabetes, placing additional pressure on remaining local NHS services. These factors all contributed to a very strained NHS context within which to introduce a new intervention. The ability to conduct contextual assessments prior to the beginning of this research study would have been helpful in preparing us for the challenges that arose relating to the primary care context. We could, for example have costed for an additional researcher time to support the implementation within practices more, or streamlined the work expected of staff from the beginning.

Another limitation of implementation within the remit of a research study is that some practices required input from the researcher in terms of providing support for the implementation, and thus making long term use of the intervention unsustainable once the research finished. Similarly, we are unable to study the ongoing implementation and maintenance of the intervention in practice as the evaluation period has finished (although the intervention is still available to patients and practices).

It is also acknowledged that we purposefully selected a CCG as the target for this research that was particularly amenable to research and had diabetes and self-management high on its agenda. Therefore, the findings from this study may not generalise to other CCGs.

The study also has several noteworthy strengths. The implementation plan development and evaluation was informed by a theory of implementation. This provided a foundation for selecting and developing the individual implementation strategies to bring about change. NPT also helped us to identify the ‘why’ of the barriers that were arising. Most of the barriers identified by staff, when analysed through an NPT lens, related to collective action. Specifically, there were such limited resources within primary care for staff to do the work of implementing HeLP-Diabetes. Using NPT allowed us to develop strategies that targeted collective action, (making the HeLP-Diabetes registration process easier to fit with the resources that were available). However, we found that NPT did not fully account for the barrier that arose with the importance staff placed on the views of patients towards HeLP-Diabetes, and as such this was not something we addressed from the outset which could have influenced the implementation.

Secondly, we consciously decided to make the implementation iterative by continually collecting data to evaluate the implementation and making adjustments to the implementation plan when needed. We found using this iterative approach particularly useful in responding to barriers that arose. This stopped ineffective practices from being continued (such as the facilitation appointment) and allowed for the deployment of new or additional strategies to be introduced to address arising problems (such as the patient self-registration process). We would argue that adopting a flexible approach is a necessity of implementation in routine practice where extensive competing demands mean unworkable practices will fall low on the list of priorities of staff.

Finally, we believe our methods were strengthened by the variety of evidence that we incorporated into the development and refinement of this implementation plan which included data from, systematic reviews, consultations key stakeholders, feedback from staff, formal interviews with staff and quantitative data.

### Generalisable learning points and implications

1. We suggest that implementation should be considered as early as possible and that much time and thought is dedicated to the process of planning an implementation strategy which draws on the best available evidence.
2. We stress that a thorough understanding of context is crucial. The implementation of an intervention should be considered before the intervention is developed (where possible), allowing the target setting and the people who will be tasked with working with it to be identified and their needs assessed which can be fed into the development and help ensure that interventions and the implementation strategies are fit for purpose.
3. We recommend the use of theory, which can be used to think through the implementation process, identify barriers and facilitators, select appropriate strategies to bring about change and to explain the implementation successes and shortcomings. We recommend that theories are selected based on defined criteria to ensure their appropriateness for the intervention and implementation context.
4. Finally, we urge those undertaking the implementation of a complex interventions in routine practice to be prepared to adopt an iterative approach the implementation: obtaining feedback from key stakeholders, responding to arising barriers and not persisting with ineffective strategies.

### Future research areas

The importance of patient factors in implementation of digital health has yet to be fully considered.

The systematic review of reviews conducted by the authors [5] found that patient related factors were rarely reported, however, one of the barriers we identified to implementing HeLP-Diabetes was staff wanting to know the views of patients towards the intervention. This may warrant further investigation; especially as digital health technologies are increasingly being designed to be used in collaboration with patients or for patient self-management. Also of importance is the issue of how best to deliver digital health interventions to patients in routine practice, given our findings that interventions that require staff support may be unworkable.

## Conclusion

This paper provides a worked example of implementing a digital health intervention within routine healthcare. The learning from this work should be of interest to others undertaking the work of planning and executing implementation activities in routine healthcare and highlights some of the challenges of this. Of particular importance is the selection of appropriate theory to guide the implementation process and strategy selection; reporting implementation strategies using clear and defined terms; ensuring enough attention is paid to planning the implementation; and a flexible approach that allows response to emerging barriers.

## Additional files

Additional file 1: Assessment of implementation theories. Assessment of sixty theories of implementation. (DOCX 58 kb)

Additional file 2: Description of the constructs and subconstructs of Normalization Process Theory. (DOCX 15 kb)

## Abbreviations

CCG: Clinical Commissioning Group
EPOC: Effective Practice and Organisation of Care
GP practice: General Practice
GPs: General Practitioners
HCA: Health Care Assistant
HCAs: Health Care Assistants
HCPs: Health Care Professionals
NHS: National Health Service
NICE: The National Institute for Health and Care Excellence
NPT: Normalization Process Theory
T2DM: Type 2 Diabetes Mellitus
