# Supplementary material for: Developing an implementation strategy for a digital health intervention: an example in routine healthcare
Source: BMC Health Serv Res. 2018 Oct 19;18:794. doi: 10.1186/s12913-018-3615-7 (PMC6194634; doi:10.1186/s12913-018-3615-7)
Supplement: Supplementary file 1 — Assessment of implementation theories. Assessment of sixty theories of implementation. (DOCX 58 kb) [file 12913_2018_3615_MOESM1_ESM.docx]

Assessment of implementation theories

| Model | Reference | Primary exclusion reason |
| --- | --- | --- |
| “4E” Framework for Knowledge Dissemination and Utilization | (Farkas and Anthony 2007) | Dissemination (rather than implementation) is sole or joint focus |
| A Conceptual Model for the Diffusion of Innovations in Service Organizations | (Greenhalgh, Robert et al. 2004) | Dissemination (rather than implementation) is sole or joint focus |
| A Conceptual Model of Knowledge Utilization | (Lester 1993) | Dissemination (rather than implementation) is sole or joint focus |
| A Convergent Diffusion and Social Marketing Approach for Dissemination | (Dearing, Maibach et al. 2006) | Dissemination (rather than implementation) is sole or joint focus |
| A Framework for Analyzing Adoption of Complex Health Innovations | (Atun, de Jongh et al. 2010) | Dissemination (rather than implementation) is sole or joint focus |
| A Framework for Spread | (Langley, Moen et al. 2009) | Dissemination (rather than implementation) is sole or joint focus |
| A Framework for the Transfer of Patient Safety Research into Practice | (Henriksen, Battles et al. 2005) | Dissemination (rather than implementation) is sole or joint focus |
| A Six-Step Framework For International Physical Activity Dissemination | (Bauman, Nelson et al. 2006) | Dissemination (rather than implementation) is sole or joint focus |
| Active Implementation Framework | (Fixsen, Naoom et al. 2005) | Doesn’t apply at system level of implementation |
| An Organizational Theory of Innovation Implementation | (Weiner, Lewis et al. 2009) | Doesn’t apply at system, community, individual or policy level of implementation |
| Availability, Responsiveness & Continuity (ARC): An Organizational & Community Intervention Model | (Glisson and Schoenwald 2005) | Doesn’t apply at system, individual or policy level of implementation |
| Blueprint for Dissemination | (Yuan, Nembhard et al. 2010) | Dissemination (rather than implementation) is sole or joint focus |
| CDC DHAP's Research-to-Practice Framework | (Collins, Harshbarger et al. 2006) | Dissemination (rather than implementation) is sole or joint focus |
| Collaborative Model for Knowledge Translation Between Research and Practice Settings | (Baumbusch, Kirkham et al. 2008) | Dissemination (rather than implementation) is sole or joint focus |
| Conceptual Framework for Research Knowledge Transfer and Utilization | (Kramer and Cole 2003) | Dissemination (rather than implementation) is sole or joint focus |
| Conceptual Model of Evidence-Based Practice Implementation in Public Service Sectors | (Aarons, Hurlburt et al. 2011) | Doesn’t apply at system, individual or policy level of implementation |
| Conceptualizing Dissemination Research and Activity: Canadian Heart Health Initiative | (Riley, Stachenko et al. 2009) | Dissemination (rather than implementation) is sole or joint focus |
| Consolidated Framework for Implementation Research | (Damschroder, Aron et al. 2009) | Doesn’t provide an explanatory framework |
| Coordinated Implementation Model | (Lomas 1993) | Dissemination (rather than implementation) is sole or joint focus |
| Critical Realism & the Arts Research Utilization Model (CRARUM) | (Kontos and Poland 2009) | Dissemination (rather than implementation) is sole or joint focus |
| Davis' Pathman-PRECEED Model | (Green and Kreuter 2005) | Dissemination (rather than implementation) is sole or joint focus |
| Diffusion of Innovation | (Rogers 2003) | Dissemination (rather than implementation) is sole or joint focus |
| Dissemination of Evidence-based Interventions to Prevent Obesity | (Dreisinger, Boland et al. 2012) | Dissemination (rather than implementation) is sole or joint focus |
| Effective Dissemination Strategies | (Scullion 2002) | Dissemination (rather than implementation) is sole or joint focus |
| Facilitating Adoption of Best Practices (FAB) Model | (Damush, Bravata et al. 2008) | Dissemination (rather than implementation) is sole or joint focus |
| Framework for Dissemination of Evidence-Based Policy | (Dodson, Brownson et al. 2012) | Dissemination (rather than implementation) is sole or joint focus |
| Framework for Knowledge Translation | (Jacobson, Butterill et al. 2003) | Dissemination (rather than implementation) is sole or joint focus |
| Framework for the Dissemination & Utilization of Research for Health-Care Policy & Practice | (Dobbins, Ciliska et al. 2002) | Dissemination (rather than implementation) is sole or joint focus |
| Framework of Dissemination in Health Services Intervention Research | (Mendel, Meredith et al. 2008) | Dissemination (rather than implementation) is sole or joint focus |
| Health Promotion Research Center Framework | (Harris 2012) | Dissemination (rather than implementation) is sole or joint focus |
| Health Promotion Technology Transfer Process | (Harris 2012) | Dissemination (rather than implementation) is sole or joint focus |
| Implementation Effectiveness Model | (Klein, Conn et al. 2001) | Doesn’t apply at system, community or policy level of implementation |
| Interacting Elements of Integrating Science, Policy, and Practice | (Meissner, Glasgow et al. 2013) | Dissemination (rather than implementation) is sole or joint focus |
| Interactive Systems Framework | (Wandersman, Duffy et al. 2008) | Dissemination (rather than implementation) is sole or joint focus |
| Knowledge Exchange Framework | (Ward, House et al. 2009) | Dissemination (rather than implementation) is sole or joint focus |
| Knowledge Translation Model of Tehran University of Medical Sciences | (Majdzadeh, Sadighi et al. 2008) | Dissemination (rather than implementation) is sole or joint focus |
| Linking Systems Framework | (Robinson, Elliott et al. 2005) | Dissemination (rather than implementation) is sole or joint focus |
| Marketing and Distribution System for Public Health | (Kreuter 2014) | Dissemination (rather than implementation) is sole or joint focus |
| Model for Improving the Dissemination of Nursing Research | (Funk, Tornquist et al. 1989) | Dissemination (rather than implementation) is sole or joint focus |
| Model for Locally Based Research Transfer Development | (Anderson, Cosby et al. 1999) | Dissemination (rather than implementation) is sole or joint focus |
| Multi-level Conceptual Framework of Organizational Innovation Adoption | (Frambach and Schillewaert 2002) | Dissemination (rather than implementation) is sole or joint focus |
| Normalization Process Theory | (May, Mair et al. 2009) | N/A |
| OPTIONS Model | (Martin, Herie et al. 1998) | Dissemination (rather than implementation) is sole or joint focus |
| Ottawa Model of Research Use | (Glasgow, Vogt et al. 1999) | Dissemination (rather than implementation) is sole or joint focus |
| Pathways to Evidence Informed Policy | (Bowen and Zwi 2005) | Dissemination (rather than implementation) is sole or joint focus |
| Policy Framework for Increasing Diffusion of Evidence-Based Physical Activity Interventions | (Owen, Glanz et al. 2006) | Dissemination (rather than implementation) is sole or joint focus |
| Practical, Robust Implementation and Sustainability Model (PRISM) | (Feldstein and Glasgow 2008) | Dissemination (rather than implementation) is sole or joint focus |
| Promoting Action on Research Implementation in Health Services (PARIHS) | (Rycroft-Malone 2004) | Doesn’t apply at system or policy level and hadn’t been applied to implementation planning |
| Pronovost's 4E's Process Theory | (Pronovost, Berenholtz et al. 2008) | Doesn’t apply at system or policy level of implementation |
| Push–Pull Capacity Model | (Green, Orleans et al. 2006) | Dissemination (rather than implementation) is sole or joint focus |
| RAND Model of Persuasive Communication and Diffusion of Medical Innovation | (Winkler, Lohr et al. 1985) | Dissemination (rather than implementation) is sole or joint focus |
| Real-World Dissemination | (Allen and Currie 2011) | Dissemination (rather than implementation) is sole or joint focus |
| Replicating Effective Programs Plus Framework | (Kilbourne, Neumann et al. 2007) | Doesn’t apply at system, individual or policy level of implementation |
| Research Development Dissemination and Utilization Framework | (Havelock, Guskin et al. 1971) | Dissemination (rather than implementation) is sole or joint focus |
| Research Knowledge Infrastructure | (Ellen, Lavis et al. 2011) | Dissemination (rather than implementation) is sole or joint focus |
| Sticky Knowledge | (Elwyn, Taubert et al. 2007) | Doesn’t apply at system or policy level of implementation |
| Streams of Policy Process | (Kingdon 1984) | Dissemination (rather than implementation) is sole or joint focus |
| The Precede–Proceed Model | (Green and Kreuter 2005) | Dissemination (rather than implementation) is sole or joint focus |
| The RE-AIM Framework | (Green and Kreuter 2005) | Dissemination (rather than implementation) is sole or joint focus |
| Utilization-Focused Surveillance Framework | (Green, Ottoson et al. 2009) | Dissemination (rather than implementation) is sole or joint focus |

**References**

Aarons, G. A., et al. (2011). "Advancing a conceptual model of evidence-based practice implementation in public service sectors." Adm Policy Ment Health **38**(1): 4-23.

Allen, B. and G. Currie (2011). "Shaping strategic change: making change in large organizations." J Health Serv Res Policy **16**(3): 184-186.

Anderson, M., et al. (1999). "The use of research in local health service agencies." Soc Sci Med **49**(8): 1007-1019.

Atun, R., et al. (2010). "Integration of targeted health interventions into health systems: a conceptual framework for analysis." Health Policy Plan **25**(2): 104-111.

Bauman, A. E., et al. (2006). "Dissemination of physical activity evidence, programs, policies, and surveillance in the international public health arena." Am J Prev Med **31**(4 Suppl): S57-65.

Baumbusch, J. L., et al. (2008). "Pursuing common agendas: a collaborative model for knowledge translation between research and practice in clinical settings." Res Nurs Health **31**(2): 130-140.

Bowen, S. and A. B. Zwi (2005). "Pathways to “Evidence-Informed” Policy and Practice: A Framework for Action." PLoS Med **2**(7): e166.

Collins, C., et al. (2006). "The diffusion of effective behavioral interventions project: development, implementation, and lessons learned." AIDS Educ Prev **18**(4 Suppl A): 5-20.

Damschroder, L. J., et al. (2009). "Fostering implementation of health services research findings into practice: a consolidated framework for advancing implementation science." Implement Sci **4**: 50.

Damush, T., et al. (2008). "Facilitation of Best Practices (FAB) Framework." Stroke QUERI Center annual report.

Dearing, J. W., et al. (2006). "A convergent diffusion and social marketing approach for disseminating proven approaches to physical activity promotion." Am J Prev Med **31**(4 Suppl): S11-23.

Dobbins, M., et al. (2002). "A Framework for the Dissemination and Utilization of Research for Health-Care Policy and Practice." Worldviews on Evidence-based Nursing presents the archives of Online Journal of Knowledge Synthesis for Nursing **E9**(1): 149-160.

Dodson, E., et al. (2012). Policy Dissemination Research. Brownson, Ross C., Graham A. Colditz, and Enola K. Proctor. Dissemination and implementation research in health: translating science to practice. Oxford University Press.

Dreisinger, M. L., et al. (2012). "Contextual factors influencing readiness for dissemination of obesity prevention programs and policies." Health Educ Res **27**(2): 292-306.

Ellen, M. E., et al. (2011). "Determining research knowledge infrastructure for healthcare systems: a qualitative study." Implementation Science **6**(1): 1-5.

Elwyn, G., et al. (2007). "Sticky knowledge: A possible model for investigating implementation in healthcare contexts." Implementation Science **2**(1): 1-8.

Farkas, M. and W. A. Anthony (2007). "Bridging science to service: using Rehabilitation Research and Training Center program to ensure that research-based knowledge makes a difference." J Rehabil Res Dev **44**(6): 879-892.

Feldstein, A. C. and R. E. Glasgow (2008). "A practical, robust implementation and sustainability model (PRISM) for integrating research findings into practice." Jt Comm J Qual Patient Saf **34**(4): 228-243.

Fixsen, D. L., et al. (2005). Implementation Research: A Synthesis of the Literature. Tampa, FL, University of South Florida, Louis de la Parte Florida Mental Health Institute, The National Implementation Research Network **(FMHI Publication #231).**

Frambach, R. T. and N. Schillewaert (2002). "Organizational innovation adoption: a multi-level framework of determinants and opportunities for future research." Journal of Business Research **55**(2): 163-176.

Funk, S. G., et al. (1989). "A model for improving the dissemination of nursing research." West J Nurs Res **11**(3): 361-372.

Glasgow, R. E., et al. (1999). "Evaluating the public health impact of health promotion interventions: the RE-AIM framework." Am J Public Health **89**(9): 1322-1327.

Glisson, C. and S. K. Schoenwald (2005). "The ARC organizational and community intervention strategy for implementing evidence-based children's mental health treatments." Ment Health Serv Res **7**(4): 243-259.

Green, L. W. and M. W. Kreuter (2005). Health program planning: An educational and ecological approach, McGraw-Hill New York.

Green, L. W., et al. (2006). "Inferring strategies for disseminating physical activity policies, programs, and practices from the successes of tobacco control." Am J Prev Med **31**(4 Suppl): S66-81.

Green, L. W., et al. (2009). "Diffusion theory and knowledge dissemination, utilization, and integration in public health." Annu Rev Public Health **30**: 151-174.

Greenhalgh, T., et al. (2004). "Diffusion of Innovations in Service Organizations: Systematic Review and Recommendations." Milbank Q **82**(4): 581-629.

Harris, J. R. (2012). "A framework for disseminating evidence-based health promotion practices." Prev Chronic Dis **9**.

Havelock, R. G., et al. (1971). Planning for innovation: through dissemination and utilization of knowledge, Center for Research on Utilization of Scientific Knowledge, Michigan (EUA).

Henriksen, K., et al. (2005). "From science to service: a framework for the transfer of patient safety research into practice."

Jacobson, N., et al. (2003). "Development of a framework for knowledge translation: understanding user context." J Health Serv Res Policy **8**(2): 94-99.

Kilbourne, A. M., et al. (2007). "Implementing evidence-based interventions in health care: application of the replicating effective programs framework." Implement Sci **2**: 42.

Kingdon, J. (1984). "Agendas, alternatives, and public policies."

Klein, K. J., et al. (2001). "Implementing computerized technology: an organizational analysis." J Appl Psychol **86**(5): 811-824.

Kontos, P. C. and B. D. Poland (2009). "Mapping new theoretical and methodological terrain for knowledge translation: contributions from critical realism and the arts." Implementation Science **4**(1): 1-10.

Kramer, D. M. and D. C. Cole (2003). "Sustained, Intensive Engagement to Promote Health and Safety Knowledge Transfer to and Utilization by Workplaces." Science Communication **25**(1): 56-82.

Kreuter, M. W. (2014). "Enhancing Dissemination Through Marketing and Distribution SystemsThree propositions about the current environment and three recommendations for improving dissemination of evidence." CREd Library **4**(1).

Langley, G. J., et al. (2009). The improvement guide: a practical approach to enhancing organizational performance, John Wiley & Sons.

Lester, J. P. (1993). "The utilization of policy analysis by state agency officials." Science Communication **14**(3): 267-290.

Lomas, J. (1993). "Retailing research: increasing the role of evidence in clinical services for childbirth." Milbank Q **71**(3): 439-475.

Majdzadeh, R., et al. (2008). "Knowledge translation for research utilization: design of a knowledge translation model at Tehran University of Medical Sciences." J Contin Educ Health Prof **28**(4): 270-277.

Martin, G. W., et al. (1998). "A social marketing model for disseminating research-based treatments to addictions treatment providers." Addiction **93**(11): 1703-1715.

May, C. R., et al. (2009). "Development of a theory of implementation and integration: Normalization Process Theory." Implement Sci **4**: 29.

Meissner, H. I., et al. (2013). "The US training institute for dissemination and implementation research in health." Implementation Science **8**(1): 12.

Mendel, P., et al. (2008). "Interventions in organizational and community context: a framework for building evidence on dissemination and implementation in health services research." Adm Policy Ment Health **35**(1-2): 21-37.

Owen, N., et al. (2006). "Evidence-based approaches to dissemination and diffusion of physical activity interventions." Am J Prev Med **31**(4 Suppl): S35-44.

Pronovost, P. J., et al. (2008). "Translating evidence into practice: a model for large scale knowledge translation." BMJ **337**.

Riley, B. L., et al. (2009). "Can the Canadian Heart Health Initiative inform the population Health Intervention Research Initiative for Canada?" Can J Public Health **100**(1): Suppl I20-26.

Robinson, K., et al. (2005). "Using linking systems to build capacity and enhance dissemination in heart health promotion: a Canadian multiple-case study." Health Educ Res **20**(5): 499-513.

Rogers, E. M. (2003). Diffusion of innovations, Simon and Schuster.

Rycroft-Malone, J. (2004). "The PARIHS Framework—A Framework for Guiding the Implementation of Evidence-based Practice." J Nurs Care Qual **19**(4): 297-304.

Scullion, P. A. (2002). "Effective dissemination strategies." Nurse Res **10**(1): 65-77.

Wandersman, A., et al. (2008). "Bridging the gap between prevention research and practice: the interactive systems framework for dissemination and implementation." Am J Community Psychol **41**(3-4): 171-181.

Ward, V. L., et al. (2009). "Knowledge brokering: exploring the process of transferring knowledge into action." BMC Health Serv Res **9**(1): 12.

Weiner, B. J., et al. (2009). "Using organization theory to understand the determinants of effective implementation of worksite health promotion programs." Health Educ Res **24**(2): 292-305.

Winkler, J. D., et al. (1985). "Persuasive communication and medical technology assessment." Arch Intern Med **145**(2): 314-317.

Yuan, C. T., et al. (2010). "Blueprint for the dissemination of evidence-based practices in health care." Issue Brief (Commonw Fund) **86**: 1-16.
