# Supplementary material for: Developing an implementation strategy for a digital health intervention: an example in routine healthcare
Source: BMC Health Serv Res. 2018 Oct 19;18:794. doi: 10.1186/s12913-018-3615-7 (PMC6194634; doi:10.1186/s12913-018-3615-7)
Supplement: Supplementary file 2 — Description of the constructs and subconstructs of Normalization Process Theory. (DOCX 15 kb) [file 12913_2018_3615_MOESM2_ESM.docx]

Description of NPT constructs and subconstructs

| **Construct** | **Coherence** | **Cognitive participation** | **Collective action** | **Reflexive monitoring** |
| --- | --- | --- | --- | --- |
| **Definition** | The **sense-making work** that people do individually and collectively when they are faced with the problem of operationalizing some set of practices. | The **relational work** that people do to build and sustain a community of practice around a new technology or complex intervention. | The **operational work** that people do to enact a set of practices. | The **appraisal work** that people do to assess and understand the ways that a new set of practices affect them and others around them. |
| ***Sub-construct*** | ***Differentiation*** | ***Initiation*** | ***Interactional workability*** | ***Systematization*** |
| **Definition** | Sense-making work to understand how a set of practices and their objects are different from each other. | Relational work to drive a set of practices forward. | The interactional work that people do with each other, with artefacts, and with other elements of a set of practices, when they seek to operationalize them in everyday settings. | Appraisal work to determine how effective and useful a set of practices are. This involves the work of collecting information in a variety of ways. |
| ***Sub-construct*** | ***Communal specification*** | ***Enrolment*** | ***Relational Integration*** | ***Communal appraisal*** |
| **Definition** | Sense-making work to build a shared understanding of the aims, objectives, and expected benefits of a set of practices. | Relational work involved in collectively contributing to the work involved in new practices. | The knowledge work that people do to build accountability and maintain confidence in a set of practices and in each other as they use them. | Appraisal work to communally evaluate the worth of a set of practices. |
| ***Sub-construct*** | ***Individual specification*** | ***Legitimation*** | ***Skill set workability*** | ***Individual appraisal*** |
| **Definition** | Sense-making work to understand specific tasks and responsibilities around a set of practices. | Relational work of ensuring that other participants believe it is right for them to be involved, and that they can make a valid contribution to it. | The allocation work that underpins the division of labour that is built up around a set of practices as they are operationalized in the real world. | Appraisal work by individuals to appraise the effects on them and the contexts in which they are set. |
| ***Sub-construct*** | ***Internalization*** | ***Activation*** | ***Contextual Integration*** | ***Reconfiguration*** |
| **Definition** | Sense-making work to understand the value, benefits and importance of a set of practices. | Relational work of participants to collectively define the actions and procedures needed to sustain a practice and to stay involved. | The resource work. Managing a set of practices through the allocation of different kinds of resources and the execution of protocols, policies and procedures. | Appraisal work by individuals or groups which may lead to attempts to redefine procedures or modify practices - and even to change the shape of a new technology itself. |
